# Better Control of Body Temperature Is Not Associated with Improved Hemodynamic and Respiratory Parameters in Mechanically Ventilated Patients with Sepsis

**DOI:** 10.3390/jcm11051211

**Published:** 2022-02-24

**Authors:** Andrej Markota, Kristijan Skok, Žiga Kalamar, Jure Fluher, Mario Gorenjak

**Affiliations:** 1 Medical Intensive Care Unit, University Medical Centre Maribor, Ljubljanska ulica 5, 2000 Maribor, Slovenia; ziga.kalamar94@gmail.com (Ž.K.); jfluher@gmail.com (J.F.); 2 Faculty of Medicine, University of Maribor, Taborska ulica 8, 2000 Maribor, Slovenia; kristijan.skok@gmail.com (K.S.); mario.gorenjak@um.si (M.G.); 3 Department of Pathology, Hospital Graz II, Location West, Göstinger Straße 22, 8020 Graz, Austria

**Keywords:** thermoregulation, targeted temperature management, sepsis, mechanical ventilation, intensive care unit, hyperthermia

## Abstract

The need for temperature modulation (mostly cooling) in critically ill patients is based on the expected benefits associated with decreased metabolic demands. However, evidence-based guidelines for temperature management in a majority of critically ill patients with fever are still lacking. The aim of our retrospective single-site observational study was to determine the differences in ICU treatment between patients in whom their temperature remained within the target temperature range for ≥25% of time (inTT group) and patients in whom their temperature was outside the target temperature range for <24% of time (outTT group). We enrolled 76 patients undergoing invasive mechanical ventilation for respiratory failure associated with sepsis. We observed no significant differences in survival, mechanical ventilation settings and duration, vasopressor support, renal replacement therapy and other parameters of treatment. Patients in the inTT group were significantly more frequently cooled with the esophageal cooling device, received a significantly lower cumulative dose of acetaminophen and significantly more frequently developed a presence of multidrug-resistant pathogens. In our study, achieving a better temperature control was not associated with any improvement in treatment parameters during ICU stay. A lower prevalence of multidrug-resistant pathogens in patients with higher body temperatures opens a question of a pro-pyrexia approach with an aim to achieve better patient outcomes.

## 1. Introduction

Fever has been recognized as a sign of illness for more than 2000 years [1]. It is highly prevalent in patients who require ICU treatment, with at least 50% of patients developing fever at some point during their ICU stay [2]. Despite this, evidence-based guidelines for targeted temperature management (TTM) in the setting of intensive care units (ICU) exist only for a fraction of patients, mostly survivors after cardiac arrest [3] and neurocritical patients [4].

For most patients who are treated in ICUs, fever is associated with infection–pyrogenic fever [1]. Traditionally, pyrogenic fever is treated at least with antipyretics and possibly with physical cooling when core body temperature (CBT) reaches around 38–38.5 °C [1]. The physiological rationale underlying temperature management in critically ill patients with fever (excluding specific patient populations) is mainly based on reduced metabolic demands associated with the reduction in basal metabolism rate after a decrease in CBT [5,6]. Therefore, the majority of temperature management interventions for this patient population are aimed at reducing CBT towards mild hyperthermia or normothermia (i.e., reduction in BT below 38–38.5 °C) [1]. Several methods can be applied to achieve a temperature decrease, usually starting with pharmacological interventions (e.g., acetaminophen), escalating to various surface cooling techniques, and, for some patients, more invasive methods [7]. Patient family members also often perceive fever as detrimental and expect fever to be managed [8], all of which lead to significant amounts of time and costs delegated to temperature management [9].

The aim of our study was to determine the differences in ICU treatment between two groups of patients: patients in whom their temperature remained within the target temperature range for ≥25% of time (inTT group) and patients in whom their temperature was outside the target temperature range for <24% of time (outTT group). Target temperatures and modalities of physical cooling were as per the treating physician.

## 2. Materials and Methods

### 2.1. Study Design and Setting

We performed a retrospective observational data collection from January 2015 to December 2019, and informed consent was waived by the institutional ethics committee (No. UKC-MB-KME-61/20). All performed procedures involving human participants were in accordance with the ethical standards of the institutional research committee and with the 1964 Helsinki declaration and its later amendments. The study was performed in a medical ICU in a tertiary level hospital.

### 2.2. Study Population

We included adult (>18 years) mechanically ventilated patients, who were intubated and mechanically ventilated because of respiratory failure associated with sepsis, and in whom physical cooling was used for temperature control (*n* = 76). We excluded patients who were treated with the targeted temperature management for accidental hypo- or hyperthermia, patients after cardiac arrest, neurocritical patients (e.g., patients with meningitis, encephalitis, ischemic or hemorrhagic stroke, subarachnoid hemorrhage or similar) and patients without a clearly defined target temperature on temperature and therapeutic charts.

Time outside the target temperature (ToTT) was defined as more than ±0.5 °C outside TT. Temperature deviation of target temperature ±0.5 °C was as per departmental policy and based on physiological basics of human thermoregulation [10,11]. No pre-specified protocol regarding target temperature or modality of physical cooling was used for this patient population—they were decided as per the treating physician. Modalities of physical cooling that were available during the study period were passive ice pads, whole body blankets with circulating water connected to a closed loop system (CritiCool, MTRE, Rehovot, Israel) and esophageal cooling devices (Esophageal Cooling Device, Advanced Cooling Therapy, Chicago, IL, USA) connected to a closed loop system (Blanketrol III, Cincinnati Sb Zero Products, Cincinnati, OH, USA).

### 2.3. Measurements

We collected basic demographic data and data relevant to ICU treatment, namely outcome of ICU treatment, ICU length of stay, modality of physical cooling, duration of physical cooling, maximum CBT during ICU stay, percentage of time above 39 °C during physical cooling, use of renal replacement therapy during physical cooling, percentage of time outside TT (defined as more than ±0.5 °C of TT), use of acetaminophen, maximum concentration of noradrenaline during physical cooling, maximum fraction of inspired oxygen (max. FiO_2_), maximum level of positive end-expiratory pressure (max. PEEP) and maximum minute ventilation (max. MV) during physical cooling, presence and location of multidrug-resistant pathogens (MDRP) during ICU stay, incidence of sacral pressure sores during ICU stay and incidence of upper gastrointestinal bleeding during ICU stay. The source data for temperature and therapeutic charts was paper-based and electronic for other data. MDRP were, according to the literature, defined as pathogens exhibiting non-susceptibility to at least one agent in three or more antimicrobial categories [12].

### 2.4. Statistical Analysis

Statistical analyses were carried out using IBM SPSS Statistics 24.0 (IBM Inc., Armonk, New York, NY, USA) and R (R Core Team 2020, Vienna, Austria). For comparison of nominal dichotomous variables, Fisher’s exact test was used. Continuous variables were first assessed for normality using D’Agostino’s omnibus test. Comparison of continuous variables across groups was carried out using Mann–Whitney U-test. Generalized linear models were used in order to additionally estimate the associations adjusted to selected covariates. Bonferroni correction was applied in order to correct for multiple comparisons of survival and hospital course parameters. A statistically significant observation was considered at P_bonferroni_ ≤ 0.05.

## 3. Results

**Figure 1:**
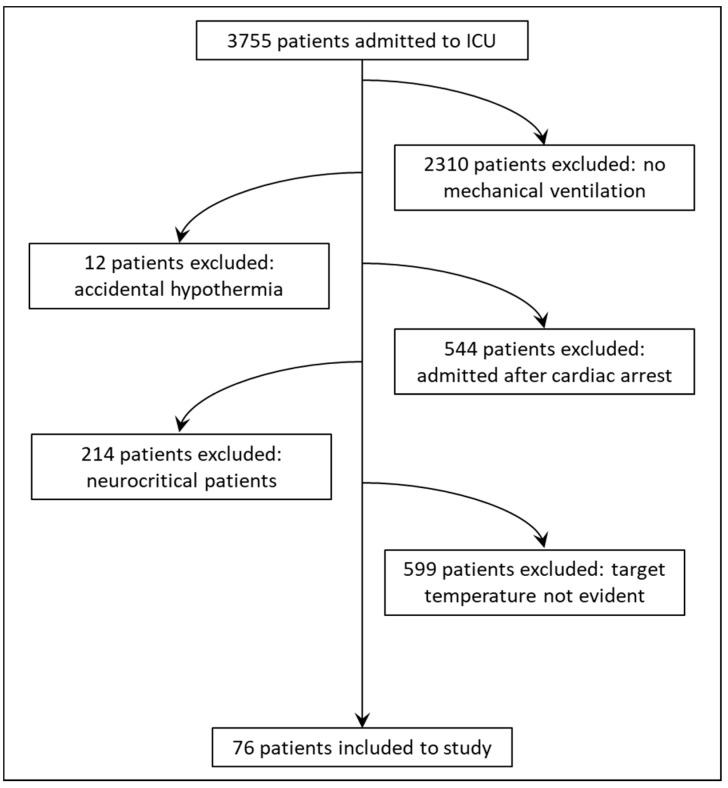
Study patient population and inclusion flowchart.

We enrolled 76 adult patients, 49 (64.5%) males and 27 females (35.5%), age 64.4 ± 12.5 years. All patients were mechanically ventilated and required noradrenaline to maintain their blood pressure with a vasoactive inotropic score of 85 ± 36.8. Twenty-seven (35.5%) patients required renal replacement therapy, and admission APACHE II score was 26.1 ± 8.3. Forty-one (53.9%) patients were discharged alive from ICU. General demographic data and parameters describing the course of the treatment in the ICU are described in Table 1. The study patient population and inclusion flowchart is presented in Figure 1.

### 3.1. Time Outside the Target Temperature Range and ICU Course of Treatment

Time outside the target temperature (ToTT) was defined as more than ±0.5 °C outside TT [10,11,13,14]. In order to assess if any differences were observable between measured parameters and ToTT, we estimated different threshold values of ToTT to obtain comparable groups of patients. Using a threshold of ≥25% for definition of ToTT, we obtained two groups consisting of 43 (57%) patients (outTT group), and 33 (43%) patients (inTT group). Using thresholds of ≥15%, ≥20%, ≥30% and ≥35%, we obtained ToTT groups consisting of 70 (92%), 59 (77%), 27 (36%) and 17 (22%), respectively. Thus, a threshold of ≥25% provided the most homogenous grouping. Subsequently, using binomial generalized linear models adjusted to sex, age, renal replacement therapy and TTM duration in days, we estimated the differences between the estimated ToTT groups as a dependent variable regarding survival and hospital course parameters (Table 2). The only statistically significant difference was observed for acetaminophen use (β: 1.62; *p* = 4.8 × 10^−3^) where larger dosages were associated with a higher probability for ToTT ≥ 25%. For all other survival and ICU treatment parameters, no statistically significant differences were observed.

### 3.2. Association between Multidrug Resistant Pathogens and Time outside the Target Temperature

We isolated MDRPs in 118 samples obtained from 36 (47.4%) patients. Most were detected in tracheal aspirates (49; 41.5%) and rectal swabs (47, 39.8%). The three most encountered MDRPs were from the genus Klebsiella (23.7%), Pseudomonas (22%) and Enterococcus (14.4%) (Table 3).

**Figure 2:**
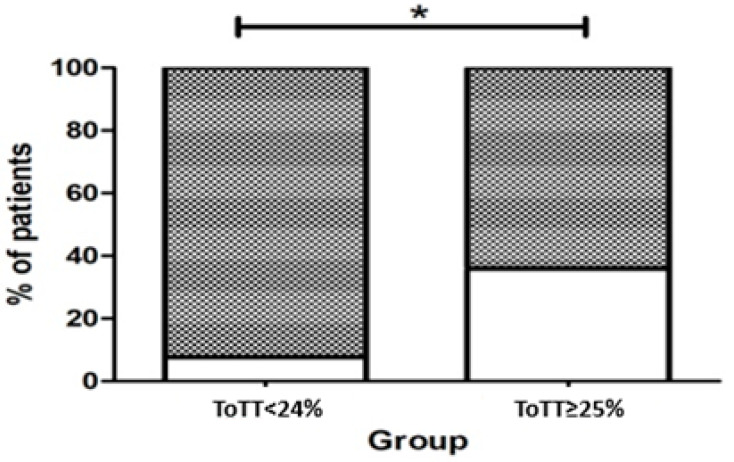
Presence of MDRP. A: % of patients with MDRP per group. *: ToTT < 24% is statistically significantly associated with a higher probability of MDRP (*p* = 0.021).

We assessed the associations of MDRP presence in relation to ToTT. We observed that patients with better controlled temperature (less ToTT) had statistically significant higher rates of MDRP presence (N: 24; 92.3%) as compared to patients with ≥25% of ToTT (N: 25; 64.1%; *p* = 0.017) (Figure 2).

To additionally confirm the findings, we performed generalized linear models, adjusted as aforementioned, and with the presence of MDRP as a dependent variable (Table 4). Using regressions, we confirmed the findings. It was shown that the ToTT < 24% is statistically significantly associated with a higher probability of MDRP (β: 2.27; *p* = 0.021).

### 3.3. Other Results

Additionally, we assessed if the modality of physical cooling in terms of ECD use exerts an impact on ToTT. Regression models were adjusted to sex, age, renal replacement therapy and TTM duration in days (Table 5). We observed that the ToTT ≥ 25% is statistically significantly associated with a lower probability of ECD use (β: −5.613; *p* = 2 × 10^−4^).

## 4. Discussion

We observed no major differences in ICU treatment parameters and outcomes between the two groups of patients apart from significantly lower use of acetaminophen, significantly greater probability of the use of esophageal cooling, and significantly higher prevalence of MDRPs in microbiology samples obtained from patients in the inTT group. To the best of our knowledge, there are no clinical studies reporting a higher prevalence of MDRPs in patients with a lower body temperature. There are several potential mechanisms that could couple body temperature with greater efficacy of antibiotics (and, hence, the lower possibility of development of MDRPs [15]) in the higher temperature group. In an animal (rabbit) model of pneumococcal meningitis, slower bacterial growth rates were observed at higher animal BT [16]. Susceptibility to antibiotics also depends on temperature, both in in vitro and in vivo setting. In in vitro setting, minimal inhibitory concentrations are decreasing inversely to increasing medium temperatures for numerous antibiotics [17]. In an animal study performed on rats [18], an increase in ertapenem concentration was observed in animals exposed to artificial warming. Similarly, increased plasma concentrations of ciprofloxacin were observed in humans who developed a higher fever [19]. In the pre-antibiotic era study [20] published in 1936, Owens reported on patients with *Neisseria gonorrhoeae* infection who were exposed to physical warming in order to achieve a body temperature of 41 °C. This treatment took around 5 h per session, usually requiring 3–4 sessions. Out of 100 patients who undertook the procedure, 64 completed the treatment and 52 were cured of gonococcal disease. Twenty-four patients refused further treatment because they did not like it and 12 could not complete the treatment because of comorbidities [20]. Similarly, treatment for dementia paralytica was performed by inoculating patients with *Plasmodium* spp., resulting in cyclic increases in body temperature, clearing syphilis in between 50 and 80% of patients, after which malaria was treated with quinine [21].

In our study, we detected no significant changes between inTT and outTT groups in duration of mechanical ventilation, levels of PEEP and FiO_2_, noradrenaline requirement and duration and ICU survival, implying that a higher level of temperature control and lower mean temperatures during the periods of targeted temperature management are not associated with clinically relevant improvements during the course of ICU treatment. A significantly lower cumulative dose of acetaminophen in the inTT group can be explained by a greater prevalence of more invasive and effective, in our case esophageal, temperature management techniques in the inTT group [22].

Similar results have been published by other authors. Young et al. performed a large, randomized, multi-center study comparing a strict temperature control strategy achieved with acetaminophen and with a placebo [23]. They achieved separation of CBT between two groups of patients (by approximately 0.5 °C), but they observed no significant differences in ICU-free days, hospital-free days, days free from mechanical ventilation, vasopressors and renal replacement therapy, and no differences in 28- or 90-day mortality. In spite of lower temperature in the acetaminophen group, they observed no differences in mean arterial pressure, heart rate and MV [23]. Similar results were reported in a recent meta-analysis of 13 randomized controlled trials that compared antipyretic with placebo in non-neurocritically ill patients. Treatment with antipyretics decreased body temperature; however, there was no observed difference in mortality [24].

Some authors suggest there is potential harm in lowering BT in septic patients. Gao et al. [25] reported a decrease in heart rate and stroke volume in patients with lower temperature, leading to a decrease in cardiac output and reduced tissue perfusion. Additionally, they reported that the levels of blood lactic acid in patients with a high temperature (>38.5 °C) were lower compared to patients with a lower temperature (<38 °C). This was explained with higher cardiac output and higher oxygen delivery in patients with a high temperature, causing increased levels of tissue perfusion and promoting the aerobic activity and function recovery of tissues and organs. Additionally, they reported a decrease in noradrenaline requirement in the higher temperature group and higher levels of proinflammatory cytokines in the higher temperature group, interpreting this as possibly beneficial for survival of patients with sepsis [25]. Similar results were reported in an animal model of sepsis, where sheep in the fever group had a higher oxygenation index, lower lactic acid level, and longer survival time [26].

There are several limitations of our study. First, we performed a small, single-center, retrospective study with inherent biases due to the design of the study. A total of 599 out of 3755 patients that were admitted to ICU during the study period were not included because relevant data (clearly defined target temperatures) could not be extracted from temperature and therapeutic charts. Second, target temperatures were as per the treating physician. However, for this patient population (i.e., general ICU patient population) there are no evidence-based guidelines to set the target temperatures. Third, various techniques and devices were used to manage temperature, such as ice pads, surface cooling with automated systems, esophageal cooling, renal replacement therapy, acetaminophen, different sedatives and opiates that interfere with normal temperature responses, etc. These factors could potentially influence our outcomes [5].

## 5. Conclusions

In our study, achieving better temperature control was not associated with any improvement of treatment parameters during the ICU stay. We discovered a lower prevalence of MDRPs in patients with higher body temperatures, which opens a question of a pro-pyrexia approach with an aim to achieve better patient outcomes.

## Figures and Tables

**Table 1:** Survival and hospital course parameters.

Characteristic (*n* = 76)	Value
Demographics, severity of illness and outcome data	
Age in years (mean ± SD)	64.4 ± 12.5
BMI kg/m2 (mean ± SD)	27.5 ± 3.9
ICU LOS days (mean ± SD)	21.8 ± 15.4
APACHE II on admission (mean ± SD)	26.1 ± 8.3
Vasoactive-inotropic score max. (mean ± SD)	85.0 ± 36.8
Outcome of death in ICU (%)	35 (46.1)
Core body temperature and temperature management data	
Temperature on admission (mean ± SD)	38.0 ± 1.0
Fever (>38.3 °C) prevalence N (%)	62 (82)
Temperature before TTM (mean ± SD)	40.0 ± 0.4
ECD N (%)	22 (28.9)
TTM duration in days (mean ± SD)	5.0 ± 1.9
Max. core body temperature during TTM (°C) (mean ± SD)	39.2 ± 0.38
% of ToTT (mean ± SD)	27.5 ± 10.1
% of time above 39 °C during TTM	16.9 ± 7.4
RRT N (%)	28 (36.8)
Procedures–pharmacological therapy	
Acetaminophen g/day (mean ± SD)	2.1 ± 0.93
Noradrenaline max dose mcg/kg/h (mean ± SD)	0.83 ± 0.23
Procedures–mechanical ventilation	
PEEP max during TTM cmH_2_O (mean ± SD)	11.5 ± 1.5
FiO_2_ max during TTM % (mean ± SD)	51.2 ± 10.8
MV max during TTM L/min (mean ± SD)	11.6 ± 2.4
Complications, multidrug resistant pathogens	
Sacral pressure sores N (%)	25 (32.9)
Upper GIT bleeding N (%)	6 (7.9)
Erythrocyte transfusion number of bags (mean ± SD)	2.8 ± 2,7
MDRP present N (%)	49 (75.4)

Legend: BMI: body mass index; ICU: intensive care unit; LOS: length of stay; APACHE II: acute physiology and chronic health evaluation score II; ECD: esophageal cooling device; TTM: targeted temperature management; ToTT: time outside target temperature; RRT: renal replacement therapy; PEEP: positive end-expiratory pressure; FiO_2_: fraction of inspired oxygen; MV: minute ventilation; MDRP: multidrug resistant pathogens.

**Table 2:** Estimation of survival and hospital course parameters regarding ToTT as dependent variable.

Parameter	β	Lower CI95	Upper CI95	*p* Value	Pbonferroni
Outcome of death (value: NO)	0.134	−0.962	1.231	0.810	1
Acetaminophen	1.62	0.747	2.49	2.8 × 10^−4^	0.00476
Noradrenaline on day 1 of TTM	−0.411	−2.209	1.387	0.654	1
Noradrenaline during TTM	0.253	−1.987	2.492	0.825	1
Noradrenaline last day of TTM	−7.275	−13.743	−0.807	0.027	0.459
PEEP on day 1 of TTM	−0.165	−0.428	0.098	0.219	1
PEEP during TTM	−0.143	−0.482	0.196	0.408	1
PEEP last day of TTM	0.020	−0.273	0.313	0.893	1
FiO_2_ on day 1 of TTM	0.005	−0.039	0.049	0.818	1
FiO_2_ during TTM	0.011	−0.039	0.061	0.663	1
FiO_2_ last day of TTM	−0.020	−0.103	0.064	0.646	1
MV max during TTM	0.131	−0.159	0.421	0.375	1
Sacral pressure sores (value: NO)	0.159	−0.928	1.245	0.775	1
GIT bleeding (value: NO)	−0.807	−2.864	1.250	0.442	1
Erythrocyte transfusion num of bags	−0.122	−0.360	0.116	0.316	1
Max core body temperature	−1.690	−3.256	−0.125	0.034	0.629
% of time above 39 °C during TTM	0.025	−0.044	0.094	0.479	1

Legend: TTM: targeted temperature management; PEEP: positive end-expiratory pressure; FiO_2_: fraction of inspired oxygen; GIT: gastro-intestinal tract; β is calculated for probability of ToTT ≥ 25%.

**Table 3:** Most common MDRP genera based on sample.

Tracheal Aspirates (n of Positive Patients = 24)												
Genus	*Klebsiella*	*Enterococcus*	*Enterobacter*	*Pseudomonas*	*Acinetobacter*	*Staphylococcus*	*Candida*	*Proteus*	*Citrobacter*	*Raoultella*	*Stenotrophomonas*	N
Number	10	4	5	11	9	2	2	1	2	2	1	49
%	20.4	8.2	10.2	22.4	18.4	4.1	4.1	2.0	4.1	4.1	2.0	
Nasopharynx swabs (n of positive patients = 23)												
Number	6	3	1	6	2	2	0	0	0	0	0	20
%	30	15	5	30	10	10	0	0	0	0	0	
Rectal swabs (n of positive patients = 23)												
Number	12	9	9	9	3	2	0	0	3	0	0	47
%	25.5	19.1	19.1	19.1	6.4	4.3	0.0	0.0	6.4	0.0	0.0	
Intravascular catheters (n of positive patients = 2)												
Number	0	1	1	0	0	0	0	0	0	0	0	2
%	0	50	50	0	0	0	0	0	0	0	0	
All samples												
Number	28	17	16	26	14	6	2	1	5	2	1	118
%	23.7	14.4	13.6	22.0	11.9	5.1	1.7	0.8	4.2	1.7	0.8	

**Table 4:** Estimation of ToTT regarding presence of MDRP as dependent variable.

Parameter	β	Lower CI95	Upper CI95	*p* Value	Pbonferroni
ToTT (binomial) (value: NO)	2.273	0.531	4.015	0.011	0.021

Legend: ToTT: time outside target temperature; β is calculated for probability of MDRP presence.

**Table 5:** Estimation of ToTT regarding ECD as dependent variable.

Parameter	β	Lower CI95	Upper CI95	*p* Value	Pbonferroni
ToTT (binomial) (value: YES)	−5.613	−8.441	−2.785	1 × 10^−4^	2 × 10^−4^

Legend: ToTT: time outside target temperature; β is calculated for probability of ECD use.

## Data Availability

The data presented in this study are available in this article. Further information or specific data are available on request from the corresponding authors.
